# Comparison of Metallization Schemes on Glass Dielectrics for X-Band Glass Antennas and Energy Harvesting

**DOI:** 10.3390/ma14237283

**Published:** 2021-11-28

**Authors:** Longzhu Cai, Qiushi Yu

**Affiliations:** 1 State Key Laboratory of Millimeter Waves, School of Information Science and Engineering, Southeast University, Nanjing 210096, China; 2 Nanjing Electronic Devices Institute, Nanjing 210016, China

**Keywords:** glass antenna, transparent antenna, conductive copper foil (CCF), conductive silver paste (CSP), indium tin oxide (ITO)

## Abstract

We prepare and test four types of glass antennas for X-band applications and energy harvesting. These antennas are made of three different glass metallization schemes, including conductive copper foil (CCF), conductive silver paste (CSP) and indium tin oxide (ITO) thin film. Compared with conventional microstrip patch antennas, the dielectric substrate materials of these designs are replaced with silicon-boron glass (*ε_r_* = 6, tangent δ = 0.002). The antenna with CCF as a radiator and ground plane (case one) is compared with the antenna with ITO replacing the radiator (case two) and ground plane (case three), respectively, and the glass antenna made of CSP (case four) is also presented. In this paper, these four types of glass antennas are measured and analyzed, and a comparison of the fabrication process and performance of these antennas is demonstrated. This study could contribute to the development of human-machine interactivity (HMI) systems with glass dielectric substrates.

## 1. Introduction

The application scenario of antennas becomes more and more diverse in form with the continuous development of mobile communication technology, which brings new requirements and challenges to antenna designs in terms of appearance and performance. In terms of performance, antennas are required to have large bandwidth, high gain, easy integration and other characteristics. In 2020, a wideband circular cavity-backed slot antenna with conical radiation patterns was shown to have a −10 dB impedance bandwidth of 16.35%, extending from 9.26 to 11.02 GHz with flat gain and conical radiation characteristics [1]. In addition, there are some other meaningful studies about antenna analysis and designs [2,3,4]. For instance, a substrate integrated waveguide planar cavity slotted antenna array was realized, and it can be excited in TE33 higher mode, which operates at X-band (10.4–10.8 GHz). This antenna array has the features of low cost and easy integration [3]. In addition to the performance of today’s antennas with a variety of features, there are also new requirements in terms of appearance. In the fields of aerospace, satellite communication, vehicle radar, etc., glass dielectric substrate-based antennas are becoming increasingly important, as it can be integrated in buildings, cars and other scenes where glass exists. Moreover, glass dielectric substrates were frequently employed as parts of the structure in human–machine interaction (HMI) systems, and the most common one is touch screen applications [5,6]. There is no doubt that the realization of communication devices (such as antennas) on glass substrates would endow these HMI systems with more functions and potentials.

One of the early attempts at using glass dielectric substrate for antenna designs was reported by scientists at the National Aeronautics and Space Administration (NASA) in 1997, and they initially verified the realizability of glass antennas in an experiment [7]. With the continuous advancement of research on glass antennas, the methods of implementing metallization on glass substrates are becoming more diverse, such as nano carbon, transparent conductive oxides, metallic nanostructures, etc. [8]. In previous studies on nano carbon based antennas, Vacirca et al. implemented a 2.4 GHz glass antenna for wireless local area network (WLAN) applications using onion-like carbon (OLC) and multi wall carbon nanotubes (MWCNT) [9]. Transparent conductive oxides (TCO) are another popular method for the realization of glass antennas, and it has been widely used in the industrial sector. Based on the used TCO structure, TCO based glass antennas can be classified as mono-layer and multilayer. In 2017, a series of glass antennas working at 28 GHz was implemented by applying four types of mono-layer TCOs, which include indium tin oxide (ITO), fluorine-doped tin oxide (FTO) and silver-coated polyester films (AgHT-4, AgHT-8) [10], respectively. These antennas can be employed on solar panels for the field of satellite communication. In a different example, multilayer TCO material was demonstrated for the use of optically transparent antenna [11]. The proposed 2.45 GHz antenna was fabricated with IZTO/Ag/IZTO multi-layer thin films, and the performance of this antenna was compared with that of antennas making a metal grid, showing acceptable characteristics [11]. Metallic nanostructure is considered as another effective technology for accomplishing glass antennas. Metallic nanostructure can be mainly divided into three forms, which are nanowire, ultra-thin metal films and patterned metal grid [8]. In 2017, a research group at Leibniz University of Hannover has successfully fabricated a glass antenna operating at 24 GHz and 61 GHz with the help of silver nanowire (AgNW) on a glass substrate [12]. The practical application of large-scale silver nanowires with high optical transparency, large electrical conductivity and strong mechanical durability is still an open problem, which was effectively solved in 2020 by a process of combining screen printing of Ag NWs with flash sintering (FLS), and the technology was used to realize the design of a new type of antenna for 5G applications [13]. Compared to nanowire, ultra-thin metal films are of higher electrical conductivity and more convenient to manipulate. In [14], the analysis of the results of a dual-band CPW-fed transparent antenna fabricated from AgHT-8 thin film indicated a strong correlation between measurement and simulation at both operating frequencies, demonstrating the usability of ultra-thin metal films for glass antenna fabrication. A significant analysis and discussion on the subject of applying metal grid for antenna applications was proposed in [15], where a glass antenna working at 24 GHz was presented. In addition, micro-copper mesh with its excellent electrical properties has a variety of applications not only in the antenna field but also in frequency selective surfaces [16]. The use of optically transparent glass substrates for energy harvesting applications is of great importance. Several antennas based on glass substrates for infrared energy harvesting applications at 30 THz was presented in [17]. In a more recent case, a broadband fully transparent antenna used for powering low power devices for the Internet of Things was studied by Nermeen A. Eltresy in 2019 [18]. In this design, glass and indium tin oxide were employed as the substrate and transparent conductor, respectively. In a different case, Evan Shi et al. investigated plain silica glass as low cost and transparent ground station substrates to reduce cost for space solar power application [19].

In this study, we further investigate the implementation of glass antennas for X-band application and energy harvesting. Four microstrip glass antennas operating at ~8 GHz were designed, fabricated and verified in experiment with various metallization techniques, including conductive copper foil (CCF), conductive silver paste (CSP) and ITO thin film. The reflection coefficients and radiation patterns of the fabricated glass antennas are compared and discussed, providing a guiding direction for the better realization of glass antennas in the future.

## 2. Materials and Methods

**Figure 1:**
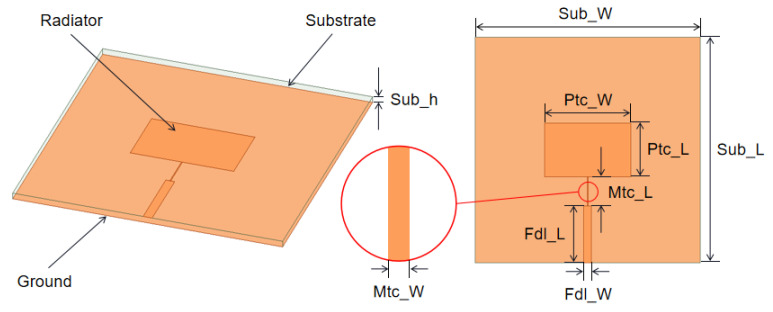
Design and dimension of micro-strip patch antenna. The image on the left is 3-dimensional structure. The image on the right is top view.

The ANSYS High Frequency Simulation Software (HFSS) (Ansys, Canonsburg, PA, USA) was employed for glass antenna design and modelling. The structure of the proposed microstrip patch antenna is depicted in Figure 1, and it is a conventional sandwich-like structure. The antenna is constructed using silicon-boron glass dielectric substrate, with its dielectric constant (*ε_r_*) and dielectric loss tangent (tangent δ) equal to 6 and 0.002, respectively, at 10 GHz and 23 °C. The glass substrate has a size of Sub_L × Sub_W × Sub_h = 30 mm × 30 mm × 0.7 mm, and it is sandwiched between the upper radiator and lower ground plane. The upper layer radiator consists of three parts: the rectangular patch, the quarter wavelength converter and the feeding line. First determine the width W of the rectangular patch, according to the following empirical formula:

(1) $$W=\frac{c}{2f_{0}}{\left(\frac{\varepsilon_{r}+1}{2}\right)}^{-1/2}$$

where *c* is the speed of light, *f*_0_ is the operating frequency and *ε_r_* is the dielectric constant of the dielectric substrate. The radiation patch length *L* is a half wavelength. Moreover, since the electromagnetic wave propagates within the medium, the guided wave wavelength *λ_e_* is defined as follows:

(2) $$\lambda_{e}=\frac{c}{f_{0}\sqrt{\varepsilon_{e}}}$$

where *ε_e_* is the effective dielectric constant, which is calculated as follows.

(3) $$\varepsilon_{e}=\frac{\varepsilon_{r}+1}{2}+\frac{\varepsilon_{r}-1}{2}{\left(1+\frac{12h}{W}\right)}^{-1/2}$$

Moreover, consider the radiation slit length Δ*L*; thus, the equivalent slit length is calculated as follows.

(4) $$\Delta L=0.412h\frac{\varepsilon_{e}+0.3}{\varepsilon_{e}-0.258}\frac{\frac{w}{h}+0.264}{\frac{w}{h}+0.8}$$

Therefore, the radiation patch length *L* is described as follows.

(5) $$L=\frac{\lambda_{e}}{2}-2\Delta L$$

After the radiation patch design is completed, the line width of the feed line needs to be considered since the microstrip feed is chosen as the feed method. The equation for the characteristic impedance *Z*_0_ of the microstrip line is described as follows:

(6) $$Z_{0}=\frac{377}{\sqrt{\varepsilon_{r}}}{\left\{\frac{w^{\prime }}{h}+0.883+0.165\frac{\varepsilon_{r}-1}{\varepsilon_{r}{}^{2}}+\frac{\varepsilon_{r}+1}{\pi \varepsilon_{r}}\left[\ln\left(\frac{w^{\prime }}{h}+1.88\right)+0.758\right]\right\}}^{-1}$$

where *w*’ is the line width of the microstrip line, and it can be observed that the impedance *Z*_0_ is related to line width *w*’, dielectric substrate thickness h and dielectric constant *ε_r_*. The above equation is written as a MATLAB program, and the corresponding data are brought into the calculation to obtain the approximate size of the desired microstrip antenna.

Set the parameters: For example, the operating frequency is 8 GHz, the thickness of the glass dielectric substrate is Sub_h is 0.7 mm and the dielectric constant is 6. The theoretical data are obtained as follows: radiation patch length Ptc_Ll is 6.4 mm, radiation patch width Ptc_W is 10 mm, feed line width Fdl_W is 1 mm and quarter wavelength Mtc_L is 4.4 mm. Since the results are calculated by empirical equations, these parameters are inputted into HFSS for modeling and optimization in order to obtain the final simulation parameters. The rectangular patch size is calculated and optimized to Ptc_L × Ptc_W = 7.1 mm × 11.5 mm. The structure in the red circle in Figure 1 is a quarter-wavelength converter for impedance matching, with a size of Mtc_L × Mtc_W = 3.9 mm × 0.1 mm. In order to realize characteristic impedance, the size of the feeding line is selected at Fdl_L × Fdl_W = 7.55 mm × 1 mm. Both radiator and ground plane are set as perfect conductors, and the optimized dimensions of each part are shown in Table 1.

**Figure 2:**
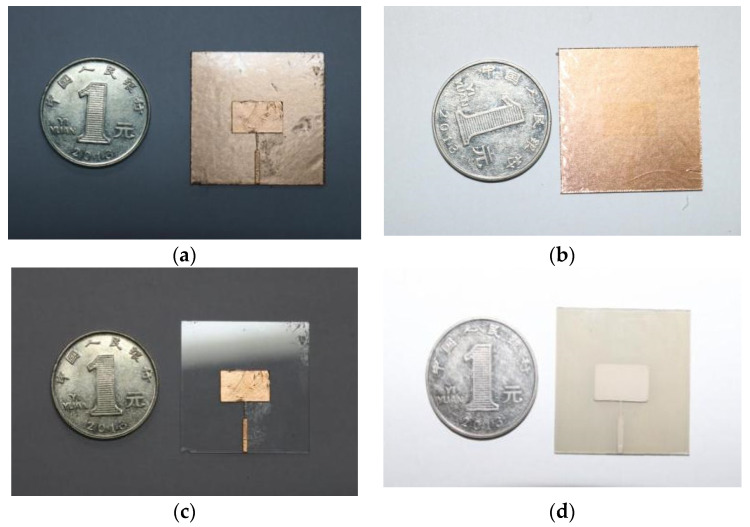
The photo of the fabricated glass antennas presented in Table 2. (**a**) Case 1. (**b**) Case 2. (**c**) Case 3. (**d**) Case 4.

Based on the above dimensions, four types of glass antennas were fabricated with three metallization techniques for top-layer radiators and bottom-layer ground planes. Three metallization techniques include using (1) conductive copper foil (CCF), (2) conductive silver paste (CSP) and (3) ITO conductive film. Table 2 illustrates the structures of these four glass antennas: (1) antenna with CCF as radiator and ground plane, as in case 1; (2) antenna with ITO film as radiator and CCF as ground plane, as in case 2; (3) antenna with CCF as radiator and ITO film as ground plane, as in case 3; and (4) antenna with CSP as radiator and ground plane, as in case 4. The photos of these fabricated samples are shown in Figure 2. The specific processing methods for the three metallization options are described in detail below.

The CCF is stuck to the glass substrate through a kind of acrylic adhesive on the back side, and the acrylic adhesive owns the properties of high-temperature resistance and chemical resistance, which is followed by the laser etching process for further treatment of CCF [20]. The laser etching machine used for processing is a picosecond UV laser (Wuhan Hero Optoelectronics Technology Co. Ltd, Wuhan, China) with a minimum focused spot of less than 10um and with a laser frequency range of 1Hz–1MHz at a wavelength of 355 nm. The laser etching machine focuses the laser onto the copper foil and heats it locally to exceed the melting point, then blows the molten metal away with high-pressure gas. The picosecond UV laser adopts the self-developed CCD automatic positioning laser etching technology, which can complete automatic etching by importing CAD data. As the laser moves relative to the copper foil on the glass substrate, the desired shape of the upper radiator can be cut out and realized. As a glass metallization solution, CCF based on laser etching technology owns the merits of high dimension precision and high electrical conductivity, while this method should also pay attention to laser control during processing in order to avoid damaging the glass substrate below or burning the required part of CCF. In this study, CCF is used in cases 1, 2, = and 3.

**Figure 3:**
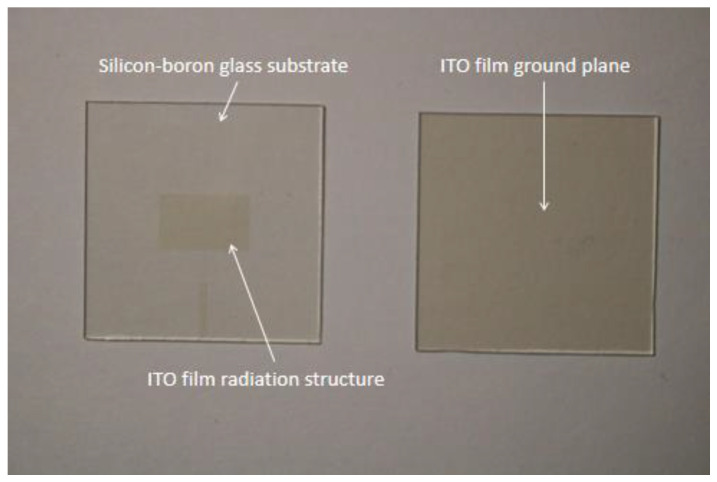
The ITO film deposited on glass. The image on the left is the top view; the image on the right is the bottom view.

**Figure 4:**
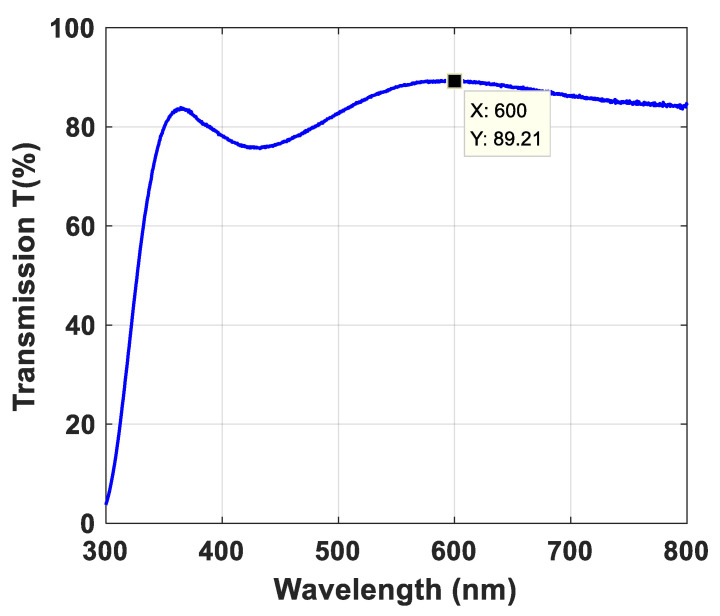
The measured transmission of the ITO film-based glass.

ITO conductive film is a semiconductor oxide material with high visible light transmission, high mechanical strength and high chemical stability, which is commonly used in industrial applications [21,22]. In cases 2 and 3, glass substrates are deposited with ITO films for realizing the radiator and ground plane by magnetron sputtering. In fabrication, ITO thin films are created by using ionized inert gas to bombard the ITO target, causing ITO atoms to be deposited on the glass substrate to form a nanoscale layer. Therefore, ITO films with high visible light transmission were initially applied in the display area, and they now can be used for the implementation of optically transparent glass antennas. The ITO film based top-layer radiator and bottom-layer ground plane are presented in Figure 3 from which we can observe that the fabricated glass sample with ITO film possesses extremely high visible light transparency. The measured transmission of ITO film-based glass at 300–800 nm wavelength is illustrated in Figure 4, and it is noticeable that the transmittance of the ITO film can reach ~90% at 600 nm.

CSP is generally a kind of conductive ink, and it is mainly composed of conductive silver powder, binder, solvent and trace additives for performance improvement. The major component of conductive silver powder is nano-silver particles with a content 60–70% [23]. In case 4, the radiation structure and ground plane of the proposed glass antenna are made of CSP. For the fabrication of the CSP-based antenna, a CSP layer was printed directly on both sides of the glass substrate with the help of printing technology. To be specific, it is an electric field driven jet-deposition micro/nano forming 3D printing technology, which is based on electrostatic field induction and electro-hydrodynamic theory. Unlike conventional electro-hydrodynamic jet 3D printing, this method only requires a conductive nozzle to be connected to a high-voltage pulsed power supply without a grounded electrode, and it could make the required electric field excited by electrostatic induction. Compared with the described laser etching technique, the printing technology is more convenient, cost effective and mature for antenna design and glass metallization. However, due to the fluidity of CSP, it might result in lower machining accuracy, and advanced processing technology and equipment are required.

In the above three metallization schemes, CCF is a kind of metallic copper layer with extremely high conductivity of $5.8\times 10^{7}\;S/m$ [24]. In contrast, CSP contains only 60–70% nano-silver, resulting in slightly lower electrical conductivity than that of the CCF. Compared with CCF and CSP, ITO films own the lowest electrical conductivity. Due to the nanoscale thickness of ITO films, the light transmission of the ITO films is much higher than that of CCF and CSP. Moreover, due to the ultra-thin property of ITO films, sheet resistance is very high. In order to analyze the performance of the three metallization schemes, a comparative experiment was conducted for these four types of antennas, as presented in the next session.

## 3. Results

### 3.1. Reflection Coefficient

**Figure 5:**
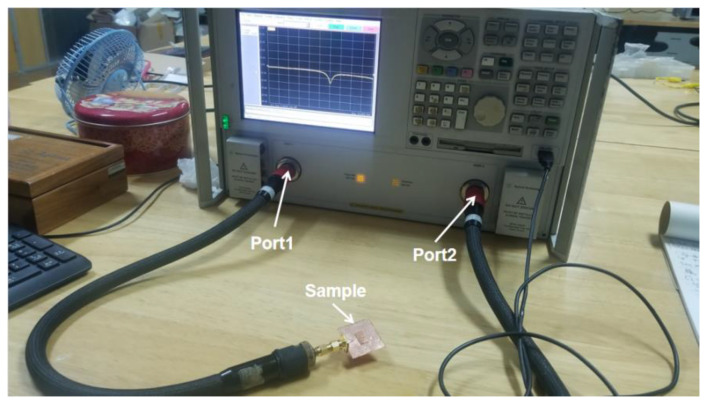
The measurement setup for antenna reflection coefficients.

**Figure 6:**
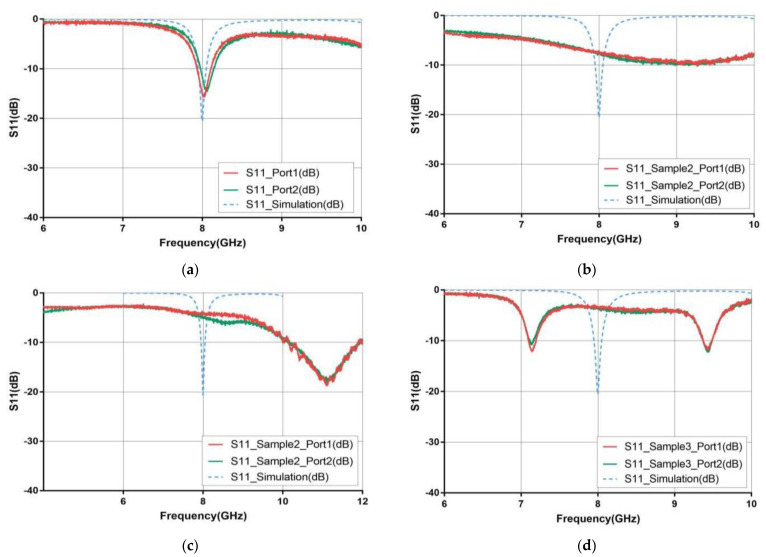
The measured and simulated reflection coefficients for the proposed four antennas. (**a**) Case 1, (**b**) case 2, (**c**) case 3 and (**d**) case 4.

The reflection coefficient of the four types of glass antennas (case 1–4) was measured by using Agilent N5230A Vector Network Analyzer (VNA) (Agilent Technologies, CA, USA). The calibration kit 3.5 mm Agilent 85052D was used for VNA calibration of both ports before measurement. The measurement setup for antenna reflection coefficient was depicted in Figure 5. The reflection coefficients of the antennas when connecting with port one and port two were tested in order to verify calibration property and reduce measurement error. Figure 6 presents the measured reflection coefficients and the corresponding simulation result. Note that all simulations of these antennas in four cases are set as follows: The boundary settings of both radiator and ground plane are ideal conductive surfaces, and the sweep range is 6–10 GHz with a step size of 0.01 GHz. Table 3 summarizes the measured and simulated results with more detail, including the resonant frequencies, the corresponding reflection coefficients and the −10 dB bandwidths of these four cases.

As observed from Table 3 and Figure 6a, the measurements of the case one antenna with CCF as the radiator and ground plane are in a good agreement with the simulation. However, the resonant frequencies of case two and case three antennas are 9.2 GHz and 11.1 GHz, with a frequency shift of 1.2 GHz (15%) and 3.1 GHz (39%), respectively, when compared with the simulated resonant frequency (8 GHz). For the case four antenna composed of CSP as the radiator and ground plane, it has two resonant frequencies at 7.15 GHz and 9.43 GHz, as shown in Figure 6d, with 0.85 GHz (11%) and 1.43 (18%) offset. For the structures of case 1–3 antennas, case two and case three are equivalent to replacing the radiator and ground plane of CCF layer in case one with ITO material, respectively. Combining the measured reflection coefficients of case two and case three antennas for analysis, the case two antenna has a smaller frequency shift, but the magnitude of the reflection coefficient at the resonant frequency for case three antenna is closer to the simulated one. Although ITO films have the advantage of highlight transmission, the best resistivity available in literature might only reach 0.4 × 10^−3^ Ω×cm [25], which is too large when comparing with metallic copper material. Therefore, the high resistivity of ITO films has an impact on the magnitude of reflection coefficients. In addition, one study presented by F. Declercq et al. implies that the semiconductor property of ITO films may affect the effective dielectric constant of the fabricated antenna [26], resulting in a shift of resonant frequency. The case two antenna uses ITO film as the radiator, and due to the aforementioned high resistivity and semiconductor property of ITO films [27], there is a frequency shift for the resonance point, and the corresponding magnitude of reflection coefficient is larger than the simulated results. A similar reason is also applicable for explaining the measured reflection coefficient of the case three antenna. While since the ground plane in case three antenna is an ITO film, the measured reflection coefficient of the case three antenna is different from that of the case 2 antenna.

**Figure 7:**
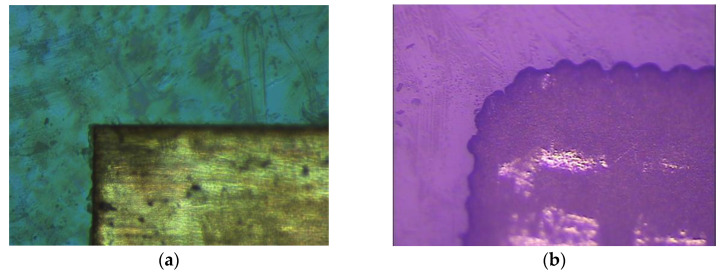
Microscopic images of the radiation patch edge. (**a**) Laser etching technology for case 1 antenna: (**b**) printing technology for case 4 antenna.

CSP material was employed for both the radiator and ground plane of the case four antenna, for which its electrical conductivity is between CCF and ITO film. We also investigated the cause of resonance shift in the case four antenna by analyzing fabrication and processing sizes. Finally, we found that the actual size of the rectangular patch width of case four antenna is 7.58 mm, which is much larger than the designed value of 7.10 mm. Figure 7 depicts the microscopic images of the radiation patch edges of the case one antenna and case four antenna, which are manipulated by laser etching technology and printing technology, respectively. It can be clearly observed from the figure that the patch edge of the case one antenna is more uniform than that of the case four antenna, and the unevenness of the case four antenna patch may be caused by the fluidity of CSP during fabrication, which results in a larger patch size than expected and, thus, affects the antenna’s performance.

### 3.2. Far-Field Radiation Pattern

**Figure 8:**
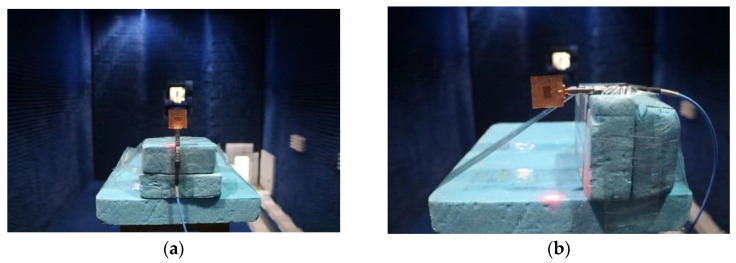
Far-field radiation pattern measurement in microwave anechoic chamber. (**a**) H-plane test. (**b**) E-plane test.

**Figure 9:**
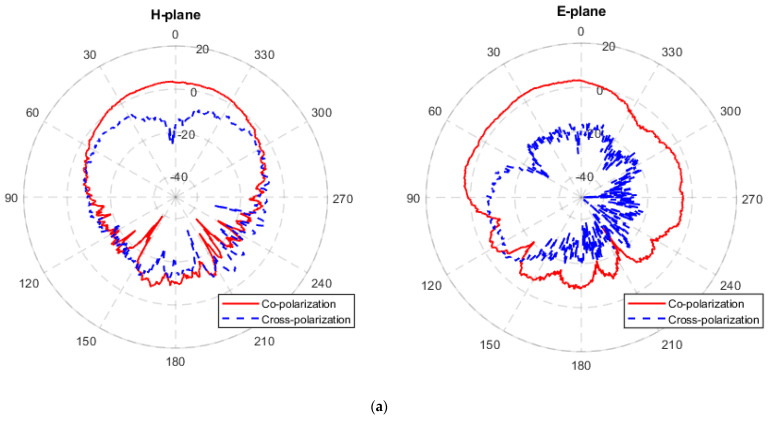

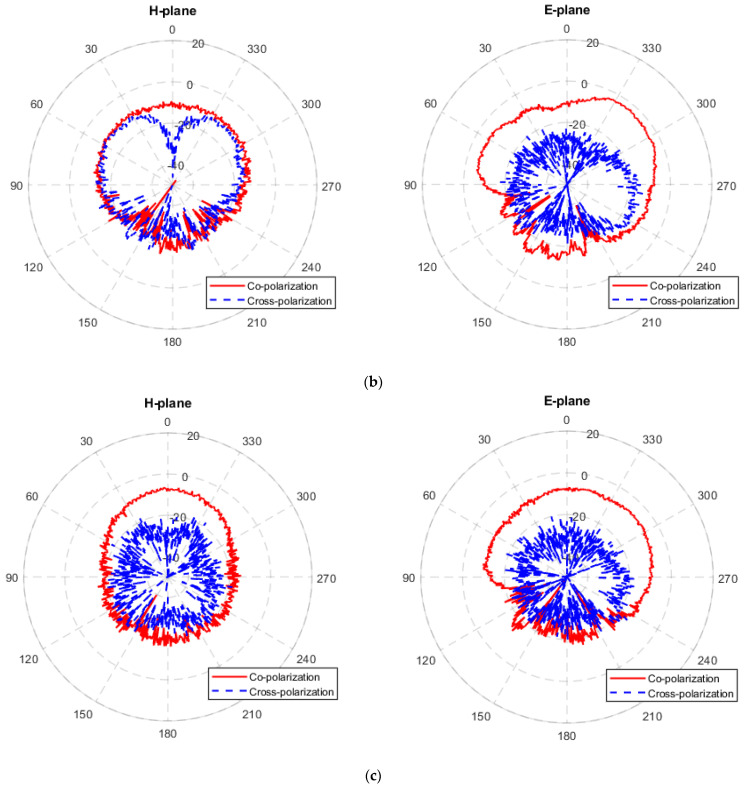

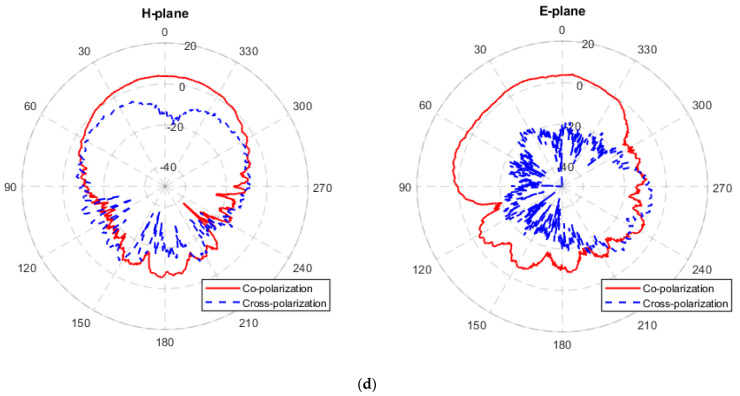
Measured H-plane and E-plane radiation pattern at center frequency. (**a**) Case 1. (**b**) Case 2. (**c**) Case 3. (**d**) Case 4. Red line: co-polarization; blue line: cross-polarization.

The far-field radiation pattern was measured in a microwave anechoic chamber. The setup for the measurement of H-plane and E-plane radiation patterns are presented in Figure 8. Standard horn antennas were applied as a reference for the normalization of radiation patterns and gains. The standard XB-GH137-20N and XB-GH90-20N horn antennas are used for the frequency bands of 5.38–8.17 GHz and 8.2–12.4 GHz, respectively. The measured H-plane and E-plane radiation patterns of these four antennas are shown in Figure 9. It is noticeable from the figure that the E-plane radiation direction diagram is a little asymmetric and deviates from the center. This is probably due to the fact that the horizontally placed antenna in E-plane measurement was not completely in the center of the rotation axis of test bench. In addition, the presence of the quarter-wavelength converter and feeding line of the side-fed microstrip glass antenna could also result in an asymmetric E-plane radiation pattern. The radiation gains of case one and case four antennas are greater than 2 dB; thus, the shapes of their radiation patterns are similar (such as H-plane). For case two and case three antennas, due to the fact that their radiation gains are lower than −4 dB, their radiation patterns are more vulnerable to measurement noise, errors and side-feeding structure, resulting in slightly different radiation patterns

The specific data of the maximum available gain (MAG) of the four antennas are shown in Table 4. Combined with Figure 9, in the four cases, the CCF glass antenna (case 1) has the best radiation performance, with a MAG of 4.60 dB at the operating frequency. In contrast, due to the high resistivity of ITO films, the MAG at the operating frequency is only −4.98 dB for the case two antenna where the radiator is replaced with the ITO structure. Similarly, case three antenna uses CCF as the radiator material, rendering its MAG slightly larger than that of the case two antenna; however, ITO films are for the ground plane, resulting in less suppression of back-scattering. As observed in Figure 9c, the forward and backward radiation pattern gains of case three is close, resulting in low MAG in the forward direction compared with case one. For the case four glass antenna composed of CSP layer, due to the better electrical conductivity of CSP material than that of ITO film, the case four antenna reveals a larger MAG. However, the conductivity of the CSP is smaller than that of the CCF, resulting in a gain of 2.10 dB for case four, which is lower than that of case one.

## 4. Conclusions

In this paper, three different glass metallization methods, including conductive copper foil (CCF), conductive silver paste (CSP) and indium tin oxide (ITO) thin film, were employed for the design of X-band glass antennas. Four glass antennas with the combination of these metallization methods were fabricated for analysis. The fabrication technique of each metallization scheme was described, and the performance of the metallization technique-based glass antennas were compared and analyzed. The maximum available gains of the case one antenna (CCF as the radiator and ground plane) and case four antenna (CSP as the radiator and ground plane) are 4.60 dB and 2.10 dB, respectively, while both of the case two (ITO as the radiator) and case three (ITO as the ground plane) antennas had much lower radiation gains due to the lossy property of ITO films [27]. This study demonstrates the feasibility of CCF and CSP as well as ITO films in the field of glass antennas. Generally, the processing accuracy of CSP needs to be improved in order to achieve better antenna design and application. The high optical transparency of ITO films is beneficial for realizing optically transparent antenna, but it is essential in enhancing antenna performance by using appropriate optimization techniques, such as multilayer film or hybrid metallization scheme.

## Figures and Tables

**Table 1:** Dimensions of the proposed glass antenna.

Structure	Length (mm)	Width (mm)	Thickness (mm)
Glass substrate	30.00	30.00	0.70
Rectangular patch	7.10	11.50	-
Quarter-wave length converter	3.90	0.10	-
Feeding line	7.55	1.00	-
Ground plane	30.00	30.00	-

**Table 2:** Composition of the 4 types of glass antennas.

Case	Top Layer	Bottom Layer	Conductor
case 1	CCF	CCF	Silicon-boron glass
case 2	ITO	CCF	Silicon-boron glass
case 3	CCF	ITO	Silicon-boron glass
case 4	CSP	CSP	Silicon-boron glass

**Table 3:** Measured and simulated resonant frequencies, the corresponding reflection coefficients and −10 dB bandwidths of the antennas.

Case	Frequency (GHz)	S11 (dB)	Bandwidth (GHz)
Simulation	8.00	−20.46	0.10
Case 1	8.01	−15.71	0.18
Case 2	9.20	−10.01	-
Case 3	11.10	−18.72	1.99
Case 4	7.15	−12.03	0.11
	9.43	−1.86	0.13

**Table 4:** Measured maximum available gain (MAG).

	Frequency (GHz)	MAG (dB)
Case 1	8.00	4.60
Case 2	9.00	−4.98
Case 3	11.00	−4.40
Case 4	7.15	2.10

## Data Availability

Data sharing is not applicable. No new data were created or analyzed in this study. Data sharing is not applicable to this article.
